# Exposure to different types of mass media and timing of antenatal care initiation: insights from the 2016 Uganda Demographic and Health Survey

**DOI:** 10.1186/s12905-022-01594-4

**Published:** 2022-01-11

**Authors:** Quraish Sserwanja, Linet M. Mutisya, Milton W. Musaba

**Affiliations:** 1 Programs Department, GOAL, Arkaweet Block 65 House No. 227, Khartoum, Sudan; 2 Maternal and Child Health Project, Swedish Organization for Global Health, Mayuge, Uganda; 3 Department of Obstetrics and Gynaecology, Busitema University, Mbale, Uganda

**Keywords:** Antenatal care, Women, Uganda, Initiation

## Abstract

**Background:**

Early initiation of antenatal care (ANC) within the first trimester is highly recommended in the current 2016 World Health Organization (WHO) guidelines. Mass media has the potential to promote early initiation of ANC because it has been used successfully in several programs. However, there is paucity of literature on the effect of exposure to different types of media on the timing of ANC initiation in Uganda. Our study aimed at exploring associations between exposure to different types of mass media and timing of ANC initiation among women in Uganda.

**Methods:**

We used a cross sectional study design, to conduct a secondary analysis of data collected in the 2016 Uganda Demographic and Health Survey (UDHS). We included weighted data of all the 10,152 women of reproductive age (15–49 years). Multistage stratified sampling was used to select study participants. Multivariable logistic regression was used to determine the association between exposure to different types of mass media and early initiation of ANC.

**Results:**

Almost a third of the women (2953/10,152, 29.1%, 95% CI 27.9–29.6) initiated their first ANC contact in the first trimester. Women who listened to radio at least once a week (adjusted OR (aOR 1.14, 95% CI 1.01–1.30) and those who watched television less than once a week (aOR 1.28, 95% CI 1.07–1.53) had higher odds of initiating ANC earlier compared to their counterparts not exposed to radio and television respectively.

**Conclusion:**

Exposure to radio and television is associated with timing of ANC initiation in Uganda. Importantly, the two types of mass media have the potential to reach women with low levels of education and encourage them to utilize maternal health services. The Ugandan government needs to prioritize and intensify the use of radio and television to promote the benefits associated with timing of ANC initiation.

**Supplementary Information:**

The online version contains supplementary material available at 10.1186/s12905-022-01594-4.

## Background

Early initiation of antenatal care (ANC) is highly recommended by the new 2016 World Health Organization (WHO) guidelines [1]. Accessing ANC services within the first trimester has been associated with better outcomes during the antenatal period, because it allows for timely identification and management of high risk patients [2, 3]. Unfortunately, there are large global disparities in ANC utilization, with sub-Saharan Africa and South Asia having the lowest utilization rates [4]. Early initiation of ANC ensures regular monitoring of the women’s health and screening for pregnancy-related complications [5]. During ANC, women receive a number of services such as micronutrient supplementation, screening and management of hypertension, immunization against tetanus, human immuno-deficiency virus (HIV) testing and counselling as part of the program for elimination of mother-to-child transmission (eMTCT) of HIV, as well as insecticide-treated mosquito nets (ITNs) plus prophylaxis against malaria [6].

ANC is an entry point to the utilization of maternal health services [7]. Studies have documented that women who initiate ANC early develop confidence in the maternity services and they are more likely to deliver under the care of a skilled birth attendant and also utilize early postnatal care (PNC); practices which have been associated with positive maternal and neonatal outcomes of pregnancy [3, 7, 8]. WHO previously recommended focused ANC Model that emphasized at least four ANC visits for every normal pregnancy [9], this was modified in 2016 from four to at least eight contacts, with the first contact to be made within the first 12 weeks of gestation [10, 11].

Over the last two decades, the utilization of maternal and child health services such as ANC in Uganda has significantly improved, but the reduction in maternal and child morbidity and mortality has not been commensurate [12]. For instance, at 64 per 1000 for under-5 mortality, 43 per 1000 for infant mortality and a maternal mortality ratio of 336 per 100,000 live birth [13], the maternal child health indicators are still unacceptably high. Although, almost all (97%) of pregnant who women in Uganda attend at least one ANC visit and 60% attend at least four visits [13], information about the early initiation of the ANC is limited. Therefore, an understanding of the level of early initiation of ANC and its determinants will inform the design of interventions to address the mismatch between the unacceptably high rates of maternal and perinatal morbidity and mortality, and the significant improvement in the utilization of maternity services.

Exposure to mass media is known to influence the utilization of maternal health services because of its ability to quickly and cheaply disseminate maternal healthcare-related and context specific information to large audiences [14]. However, what remains unclear is the effect of exposure to different types of mass media and the uptake of maternal health services in Uganda. Earlier studies on the timing of ANC and mass media had some limitations, for instance Bbaale et al. only looked at effect of exposure to radio [15], while Atuhaire et al. explored the effect of exposure to mass media as a composite variable [7]. Therefore, understanding the association between timing of ANC initiation and the exposure to the different types of mass media is crucial because access to them varies across regions, wealth indices and age groups. In addition, the different types of mass media appeal to different segments of the population on the basis of several factors such as age, level of education and packaging of the messages. Hence our study aimed at exploring associations between exposure to the different types of mass media and timing of antenatal care initiation among women in Uganda using the 2016 UDHS data.

## Methods

### Study design

This was a nationally representative cross-sectional study. We conducted a secondary data analysis of the 2016 Uganda demographic health survey (UDHS) data. The UDHS collected data on women's sociodemographic characteristics, reproductive health and nutrition indicators [16, 17]. The UDHS data were collected from June to December 2016 [13] implemented by the Uganda Bureau of Statistics (UBOS) with the technical assistance of Inner City Fund (ICF) International through the USAID-supported MEASURE DHS project [13]. These data were obtained at two-stage cluster sampling. Selection of cluster sample was done at the first stage and then selection of households at the second stage. UDHS 2016 included women aged 15 to 49 years who were either permanent residents or slept in the selected household the night before the survey [13]. In this study, we included women aged 15–49 years who had a live birth within five years preceding the survey and had given informed consent. The UDHS interviewed 18,506 women aged 15–49 years of which 10,152 had a live birth within five years preceding the survey [13].

### Outcome variable

The primary outcome was early initiation of ANC defined as timing of first ANC contact within the first trimester of pregnancy coded as one and initiation after the first trimester coded as zero [18].

### Exposures

Women were asked whether they read a newspaper or magazine, listen to radio or watch TV almost every day, at least once a week, less than once a week or not at all [13]. Responses were available from only at least once a week, less than once a week and not at all.

### Covariates

We included determinants of timing for ANC initiation based on the available literature and data [2, 4, 5]. At community level, the variables included were the place of residence (rural and urban), and the region of residence (Central, East, West and North). The household level variables considered were household size (less than six and six), sex of household head (male and female), and above the wealth index that was categorized into quintiles that ranged from the poorest to the richest quintile. We considered the following individual level factors; age (15–24, 25–34 and 35–49), parity (less than 4 and above 4), working status (yes and no), marital status (married and not married), education level of the women and the partner (no education, primary, secondary and tertiary).

### Analytic approach

In order to account for the unequal probability sampling in different strata and to ensure representativeness of the study results, we applied DHS sample weights [19, 20]. Furthermore, we used SPSS version 25.0 statistical software (Armonk, NY: IBM Corp) complex samples package incorporating the following variables in the analysis plan to account for the multistage sample design inherent in the DHS dataset: individual sample weight, sample strata for sampling errors/design, and cluster number [19–21]. Use of complex samples package ensures that the sample design is incorporated into the analysis leading to accurate and reliable results. Tabulation for independent variables was done for proportions and frequencies. Bivariable logistic regression was done to assess the association of each independent variable with timing of ANC initiation and crude odds ratio (COR), 95% confidence interval (CI) and p-values are presented. Two multivariable logistic regression models were made based on complete case samples with the first one having mass media variables and the final one having other sociodemographic independent variables that were found significant at bivariable level (p-value < 0.25) [22]. The second model determined whether the association between mass media and timing of ANC initiation remains statistically significant while controlling for other sociodemographic factors. Adjusted odds ratios (AOR), 95% Confidence Intervals (CI) and p-values were calculated with statistical significance level set at p-value < 0.05. All variables in the model were assessed for collinearity, and the highest variance inflation factor (VIF) was 2.24 [23]. Sensitivity analysis was done after removing the variable with missing data (partner’s level of education) in the second multivariable model as shown in Additional file 1.

## Results

Table 1 shows the descriptive characteristics of study participants. Regarding exposure to mass media, listening to radio had the highest prevalence at 73.7% following by watching TV at 29% and the least prevalence was observed with reading newspapers at 19.3%. Most study participants were residing in rural areas (76.9%), were working (79.0%), married (81.3%) and belonged to households that were headed by a male (73.1%). Almost a third of the women (29.1%, 95% CI 27.9–29.6) initiated their first ANC contact in the first trimester.

**Table 1:** Background characteristics of Ugandan women aged 15 to 49 years as per the 2016 UDHS

Category	Frequency (N = 10,152)	Percent (%)
**Age**		
15–24	3546	34.9
25–34	4425	43.6
35–49	2181	21.5
**Residence**		
Urban	2346	23.1
Rural	7807	76.9
**Region**		
Western	2559	25.2
Eastern	2727	26.9
Central	2805	27.6
Northern	2061	20.3
**Parity**		
0–4	6699	66.0
5 and above	3453	34.0
**Household size**		
6 and Above	5062	49.9
Less than 6	5090	50.1
**Working status**		
Not working	2136	21.0
Working	8016	79.0
**Marital status**		
Married	8256	81.3
Not married	1896	18.7
**Education level**		
No education	1061	10.5
Primary education	6091	60.0
Secondary education	2285	22.5
Higher	715	07.0
**Wealth index**		
Poorest	2117	20.9
Poorer	2074	20.4
Middle	1921	18.9
Richer	1862	18.3
Richest	2178	21.5
**Exposure to radio**		
Not at all	2668	26.3
Less than once a week	1551	15.3
At least once a week	5933	58.4
**Exposure to newspapers**		
Not at all	8188	80.6
Less than once a week	1209	11.9
At least once a week	755	7.4
**Exposure to TV**		
Not at all	7211	71.0
Less than once a week	1105	10.9
At least once a week	1836	18.1
**Husband’s education level**^**a**^		
None	517	5.1
Primary	4346	42.8
Secondary	2205	21.7
Tertiary	975	9.6
**Sex of household head**		
Female	2726	26.9
Male	7426	73.1
**Timing of ANC**		
1–3 months	2953	29.1
Above 3 months	7199	70.9

^a^Missing 2109 women

### Exposure to different types of mass media and timing of ANC initiation

In the final adjusted model (Table 2), women who listened to radio at least once a week were 14% more likely to initiate ANC contacts in the first trimester compared to those who did not listen to radio (aOR 1.14, 95% CI 1.01–1.30). Women who watched TV less than once a week were 28% more likely to initiate ANC contacts in the first trimester compared to those who did not watch TV (aOR 1.28, 95% CI 1.07–1.53). Region, age of the women and household size were also associated with timely initiation of ANC contacts. Women in Northern and Western Uganda, those belonging in households with less than five members, aged 25–34 years were more likely to initiate ANC contacts in the first trimester compared to those in Eastern Uganda, those in households with five and above members and those aged 35–49 years respectively.

**Table 2:** Associations between exposure to different types of mass media and timing of ANC initiation in Uganda

Characteristics	Crude model COR (95%CI)	P-value	Adjusted model I AOR (95%CI)	Adjusted model II AOR (95% CI)
**Exposure to radio**		0.045		
Not at all	1		1	1
Less than once a week	**1.22 (1.04–1.42)**		**1.18 (1.01–1.39)**	1.13 (0.94–1.35)
At least once a week	1.09 (0.98–1.22)		1.06 (0.95–1.19)	**1.14 (1.01–1.30)**
**Exposure to newspapers**		0.265		
Not at all	1		1	1
Less than once a week	1.06 (0.90–1.25)		1.03 (0.87–1.21)	0.98 (0.80–1.19)
At least once a week	1.17 (0.95–1.45)		1.19 (0.94–1.49)	1.11 (0.85–1.45)
**Exposure to television**		0.025		
Not at all	1		**1**	**1**
Less than once a week	**1.25 (1.06–1.47)**		**1.20 (1.02–1.42)**	**1.28 (1.07–1.53)**
At least once a week	1.01 (0.87–1.16)		0.96 (0.82–1.12)	1.12 (0.91–1.37)
**Region**		< 0.001		
East	1			**1**
North	**1.70 (1.44–2.00)**			**1.61 (1.35–1.94)**
West	**1.59 (1.35–1.86)**			**1.52 (1.27–1.83)**
Central	1.09 (0.91–1.30)			0.92 (0.73–1.15)
**Residence**		0.716		
Urban	1			
Rural	0.97 (0.84–1.28)			
**Working status**		0.141		
Working	1			1
Not working	0.89 (0.78–1.04)			0.99 (0.85–1.15)
**Marital status**		0.783		
Not married	1			
Married	0.98 (0.87–1.11)			
**Education level**		< 0.001		
Tertiary	1			1
Secondary Education	**0.64 (0.50–0.82)**			0.75 (0.56–1.00)
Primary Education	**0.66 (0.53–0.83)**			0.80 (0.58–1.09)
No Education	0.81 (0.63–1.05)			0.89 (0.62–1.27)
**Wealth Index**		0.960		
Richest	1			
Richer	0.98 (0.82–1.18)			
Middle	0.99 (0.84–1.17)			
Poorer	0.95 (0.79–1.13)			
Poorest	0.96 (0.81–1.14)			
**Age (years)**		< 0.001		
35–49	**1**			**1**
25–34	**1.32 (1.16–1.49)**			**1.21 (1.02–1.44)**
15–24	**1.19 (1.04–1.35)**			1.13 (0.91–1.39)
**Household size**		0.004		
Above 5	1			1
Less than 5	**1.16 (1.05–1.29)**			**1.17 (1.03–1.33)**
**Parity**		0.001		
Above 4	1			1
Less than 4	**1.22 (1.10–1.36)**			1.07 (0.90–1.27)
**Sex of household head**		0.783		
Male	1			
Female	0.98 (0.88–1.11)			
**Age at first birth**		0.169		
18 and above	1			1
Less than 18	0.93 (0.84–1.03)			1.05 (0.94–1.18)
**Partner’s education**	148	< 0.001		
Tertiary	1			1
Secondary	**0.77 (0.63–0.93)**			0.89 (0.72–1.09)
Primary	**0.72 (0.59–0.86)**			0.83 (0.67–1.02)
No education	1.03 (0.79–1.35)			1.13 (0.85–1.51)

Bold significant at P-value less than 0.05

## Discussion

This current study assessed the association between exposure to the different types of mass media and timing of ANC initiation. We found that exposure to both radio and television were associated with early initiation of ANC. In addition, region, age and household size were also associated with timely initiation of ANC. Overall, radio is the most popular media source compared with television and newspapers/magazines. Most people depend on listening to radio as the primary source of information especially in rural areas of Africa [24]. Not only is it widespread and popular, but it’s also more accessible, convenient, inexpensive in that it’s a cost-effective way to share information with a large audience, capable of delivering information quickly and interactive where listeners are allowed to ask questions and give feedback [24]. It should be noted that most of women in this study were resident in the rural areas (79.9%) compared to those in urban areas (23.1%). Similar to most sub-Saharan settings, public health facilities in rural areas that provide free services are usually less, hence women have to cover long distances. In addition, rural health facilities are under staffed which negatively affect access and utilization of maternal health services [25, 26]. In such settings, where reaching all mothers by health workers is not possible, mass media remains a feasible option to reaching wider audiences to disseminate message on the importance of timely ANC and availability of such service in their own communities [2].

The association between both listening to radio, watching television and early initiation of ANC is not surprising because similar studies from various low resource settings such as Ethiopia and Malawi have reported this positive association [27–30]. Women who were exposed to radio at least once a week were more likely to initiate ANC within the first trimester compared to those who were not exposed to radio at all. Women who were exposed to television less than once a week were more likely to initiate ANC within the first trimester compared to those who were not exposed at all. These findings are consistent with a study conducted in Ethiopia [30] which showed that women who had television or radio exposure, were two times more likely to initiate ANC within the recommended time. Mass media including television and radio lead to positive healthy behavioral change through regular and frequent broadcasted programs and announcements that inform masses about the benefits of timely initiation of ANC and other maternity care services [5]. They also inform the masses about the availability and working hours of public health facilities that provide free services. This information motivates women and their partners to take practical action towards their health [5]. Similarly, in Nepal 60% and 43.1% of respondents from the rural community were reported to have exposure to radio and television respectively [2]. These two types of mass media had positive effect on utilization of ANC components such as frequency of ANC visits [2]. India and South Asia also reported positive association of mass media and utilization of maternal health services at all the three critical stages of pregnancy, however they did not go further to establish the effect of the association [4, 5].

Exposure to newspaper or magazines was not associated with timing of ANC initiation in this study. This could partly be explained by that most women reside in rural areas where access to Newspapers or magazines is hard and not sustainable due to poor road network to ensure regular supply chain and the daily or weekly costs involved in purchasing them [31]. Furthermore, with over 70% of women in this study having primary level of education and below indicate low literacy levels which negatively affect utilization of newspapers or magazines. The other factors associated with early initiation of ANC included; women of younger age, less household size and Northern region. Socio-economic variables have an influence on timely initiation of ANC by affecting how these mass media messages are received and utilized by women. For instance, women aged between 25 and 34 years were more likely to initiate ANC within the recommended time compared to the older women. A similar finding has been reported elsewhere [30]. The younger women are more likely to listen to radio and watch television for edutainment. Consequently, younger mothers tend to be more knowledge about modern maternal healthcare services than their older counterparts who tend to rely a lot on their experience of maternal health [5, 32, 33]. This has the potential to negatively affect their utilization of maternal health services that are promoted on mass media. Women in larger households were less likely to initiate ANC in the first trimester as they are more likely to have more household chores and responsibilities such as taking care of children, cooking and cleaning, hence tend to have less time for their health matters [34] and to listen to mass media programs.

Women from the Northern and Western regions had higher odds of earlier timing of ANC initiation compared to the Eastern region. Similarly, a 2011 UDHS study by Rutaremwa et al. showed that although other regions had less odds of utilizing maternity care services compared to Kampala, utilization odds of the desirable maternal health services package was highest with the Northern region compared to other regions [35]. The Northern region experienced a long civil war compared to the other regions, keeping internally displaced people in camps longer. During and after the civil war, radio was the common source of information listened by a large audience [36–38]. Presence of a large number of people staying in camps with increased utilization of radio and limited or no movements made it easier for humanitarian organizations to implement several interventions and programs, especially those that targeted maternal health services. These services became more accessible to women in the camps during and after the civil war, hence the observed finding in the region [39, 40]. Besides being one of the poorest region in Uganda, a study by Tetui et al. that focused on health system factors in the region showed significant staff shortage with insufficient medical supplies and inadequate ANC package [41]. These factors could partly lead to poor patient satisfaction in Eastern Uganda leading to delayed ANC initiation.

Finally, the widespread accessibility and use of the internet has increasingly enabled women to independently seek out pregnancy-related information, social and emotional support during the antenatal period [42]. Social media and mHealth apps are increasingly being used because of better access to internet services [43]. Unfortunately, social media was not studied in this UDHS data set. However, it has got the potential to supersede the other forms of traditional mass media because people will rather pay for a subscription for an internet connection than buy a newspaper. Dekker et al. showed how important social media is in finding and disseminating information about evidence-based maternity care. This study showed women to have been highly engaged in using social media to find and share maternity information with the majority having the intention to use this information [44]. The absence of social media data in DHS and the dearth of evidence of association between social media and maternal healthcare utilization raises concerns about the unknown benefits and risks of finding, using, and sharing maternity information on different social media platforms. The link between social media and health care is continuously evolving, hence DHS should consider adding social media in the subsequent surveys.

### Strengths and limitations

A strength of this study is that it’s the first study to assess the association between exposure to different types of mass media and timing of ANC with a high response rate of 97%. Secondly, we used a nationally representative sample, therefore, the findings are generalizable to all women in Uganda. The study was however a cross-sectional survey that restricts the interpretation of causality. UDHS did not collect information on exposure to social media, future surveys should consider including it among the variables under mass media. Lastly, the dataset did not include information about the content of mother's mass media exposure.

## Conclusion

Exposure to radio and television is associated with timing of ANC initiation in Uganda. Importantly, the two types of mass media have the potential to reach women with low levels of education and encourage them to utilize maternal health services. Major implications emanating from this study include the need for targeted interventions to improve utilization of maternal services that are specific to regional needs, taking advantage of radio and television. We recommend the Ministry of Health to allocate more resources to radio and television segments of media for its maternal and child health related awareness campaigns in the country. There is need to target older women with level of education below tertiary, residing in the Eastern region and from larger households.

## Supplementary Information

**Additional file 1.** Associations between exposure to different types of mass media and timing of ANC initiation in Uganda.

## Data Availability

The data set used is openly and freely available upon permission from MEASURE DHS website (URL: https://www.dhsprogram.com/data/available-datasets.cfm).

## Abbreviations

AOR: Adjusted odds ratio
CI: Confidence interval
COR: Crude odds ratio
DHS: Demographic Health Survey
UDHS: Uganda Demographic Health Survey
OR: Odds ratio
SD: Standard deviation
WHO: World Health Organization
SPSS: Statistical Package for Social Science
USAID: United States Agency for International Development.
ANC: Antenatal care
TV: Television
