# Supplementary material for: Exposure to different types of mass media and timing of antenatal care initiation: insights from the 2016 Uganda Demographic and Health Survey
Source: BMC Womens Health. 2022 Jan 11;22:10. doi: 10.1186/s12905-022-01594-4 (PMC8751065; doi:10.1186/s12905-022-01594-4)
Supplement: Supplementary file 1 — Additional file 1. Associations between exposure to different types of mass media and timing of ANC initiation in Uganda. [file 12905_2022_1594_MOESM1_ESM.docx]

**Exposure to different types of mass media and timing of antenatal care initiation: Insights from the 2016 Uganda Demographic and Health Survey**

**Supplementary file 1: Associations between exposure to different types of mass media and timing of ANC initiation in Uganda**

| **Characteristics** | **Crude model**  **COR (95%CI)** | **P-value** | **Adjusted model I**  **AOR (95%CI)** | **Sensitivity model**  **AOR (95% CI)** |
| --- | --- | --- | --- | --- |
| **Exposure to Radio** |  | 0.045 |  |  |
| Not at all | 1 |  | 1 | 1 |
| Less than once a week | **1.22 (1.04-1.42)** |  | **1.18 (1.01-1.39)** | **1.17 (1.01-1.37)** |
| At least once a week | 1.09 (0.98-1.22) |  | 1.06 (0.95-1.19) | 1.09 (0.97-1.23) |
| **Exposure to Newspapers** |  | 0.265 |  |  |
| Not at all | 1 |  | 1 | 1 |
| Less than once a week | 1.06 (0.90-1.25) |  | 1.03 (0.87-1.21) | 1.07 (0.91-1.28) |
| At least once a week | 1.17 (0.95-1.45) |  | 1.19 (0.94-1.49) | 1.20 (0.95-1.50) |
| **Exposure to television** |  | 0.025 |  |  |
| Not at all | 1 |  | **1** | **1** |
| Less than once a week | **1.25 (1.06-1.47)** |  | **1.20 (1.02-1.42)** | **1.27 (1.07-1.50)** |
| At least once a week | 1.01 (0.87-1.16) |  | 0.96 (0.82-1.12) | 1.08 (0.90-1.28) |
| **Region** |  | <0.001 |  |  |
| East | 1 |  |  | **1** |
| North | **1.70 (1.44-2.00)** |  |  | **1.68 (1.43-1.97)** |
| West | **1.59 (1.35-1.86)** |  |  | **1.55 (1.32-1.82)** |
| Central | 1.09 (0.91-1.30) |  |  | 0.95 (0.78-1.15) |
| **Residence** |  | 0.716 |  |  |
| Urban | 1 |  |  |  |
| Rural | 0.97 (0.84-1.28) |  |  |  |
| **Working status** |  | 0.141 |  |  |
| Working | 1 |  |  | 1 |
| Not working | 0.89 (0.78-1.04) |  |  | 0.95 (0.82-1.10) |
| **Marital status** |  | 0.783 |  |  |
| Not Married | 1 |  |  |  |
| Married | 0.98 (0.87-1.11) |  |  |  |
| **Education Level** |  | <0.001 |  |  |
| Tertiary | 1 |  |  | 1 |
| Secondary Education | **0.64 (0.50-0.82)** |  |  | **0.69 (0.53-0.89)** |
| Primary Education | **0.66 (0.53-0.83)** |  |  | **0.70 (0.54-0.91)** |
| No Education | 0.81 (0.63-1.05) |  |  | 0.85 (0.63-1.16) |
| **Wealth Index** |  | 0.960 |  |  |
| Richest | 1 |  |  |  |
| Richer | 0.98 (0.82-1.18) |  |  |  |
| Middle | 0.99 (0.84-1.17) |  |  |  |
| Poorer | 0.95 (0.79-1.13) |  |  |  |
| Poorest | 0.96 (0.81-1.14) |  |  |  |
| **Age (years)** |  | <0.001 |  |  |
| **35-49** | **1** |  |  | **1** |
| **25-34** | **1.32 (1.16-1.49)** |  |  | **1.24 (1.07-1.44)** |
| 15-24 | **1.19 (1.04-1.35)** |  |  | 1.12 (0.93-1.26) |
| **Household size** |  | 0.004 |  |  |
| Above 5 | 1 |  |  | 1 |
| Less than 5 | **1.16 (1.05-1.29)** |  |  | 1.11 (1.00-1.24) |
| **Parity** |  | <0.001 |  |  |
| Above 4 | 1 |  |  | 1 |
| Less than 4 | **1.22 (1.10-1.36)** |  |  | 1.10 (0.94-1.29) |
| **Sex of household head** |  | 0.783 |  |  |
| Male | 1 |  |  |  |
| Female | 0.98 (0.88-1.11) |  |  |  |
| **Age at first birth** |  | 0.169 |  |  |
| 18 and above | 1 |  |  | 1 |
| Less than 18 | 0.93 (0.84-1.03) | 55  5  66  3f  444  888 |  | 1.02 (0.92-1.13) |
| **Partner’s education**  **N** | 148 | <0.001 |  |  |
| Tertiary  Primary | 1  1309 |  |  | - |
| Secondary | **0.77 (0.63-0.93)** |  |  |  |
| Primary | **0.72 (0.59-0.86)** |  |  |  |
| No education | 1.03 (0.79-1.35) |  |  |  |

**Bold** significant at p-value less than 0.05
